# Supplementary material for: Feeding Cows with Olive Cake Enriched in Polyphenols Improves the Sustainability and Enhances the Nutritional and Organoleptic Features of Fresh Caciocavallo Cheese
Source: Foods. 2024 Oct 18;13(20):3320. doi: 10.3390/foods13203320 (PMC11508000; doi:10.3390/foods13203320)
Supplement: Supplementary file 1 [file foods-13-03320-s001.zip › foods-3234644-supplementary.pdf]

**Table S1.** Ingredients and chemical composition of dietary treatments, Olive Cake and Hay. CTR treatment: concentrate without olive cake supplements. ECO treatment: concentrate with 7% enriched olive cake as 7% of a DM.

| | Treatment | | | |
|---|---|---|---|---|
| **Ingredients, % of DM** | **CTR** | **ECO** | **Olive Cake** | **Grass hay** |
| Enriched Olive Cake | - | 7 | | |
| Corn meal | 40 | 41 | | |
| Roasted soybean flour | 16.5 | 17 | | |
| Barley meal | 12 | 10 | | |
| Beetpulp | 9 | 3 | | |
| Wheat bran | 6 | 7 | | |
| Sunflower meal. | 6 | 5.5 | | |
| RUMEN Bypass Fat | 2.5 | 1.5 | | |
| Minerals and Vitamins Mix | 1.5 | 1.5 | | |
| Calcium Carbonate | 1.3 | 1.3 | | |
| Saccharomyces dried yeast | 1 | 1 | | |
| Cane molasses | 1 | 1 | | |
| Na bicarbonate | 1 | 1 | | |
| Na chloride | 0.7 | 0.7 | | |
| P dicalcium | 0.6 | 0.6 | | |
| NutriGen 40 C | 0.5 | 0.5 | | |
| Mg oxide | 0.4 | 0.4 | | |
| Chemical composition, % of DM | | | | |
| CP | 17.7 | 18.11 | 11.23 | 6.77 |
| Fat | 5.01 | 5.05 | 16.44 | 1.37 |
| Starch | 45.64 | 38.70 | 15.13 | |
| Crude Fiber | 4.40 | 6.02 | | |
| NDF | | | 59.40 | 59.38 |
| ADF | | | 48.46 | 41.08 |
| ADL | | | 22.48 | 6.89 |
| Ash | 9.84 | 9.78 | 4.35 | 6.81 |
| Nel, Mcal/kg of DM | 1.81 | 1.85 | | |
| Total polyphenols, ppm | 566.0 | 782.36 | 9,350.00 | 344.18 |

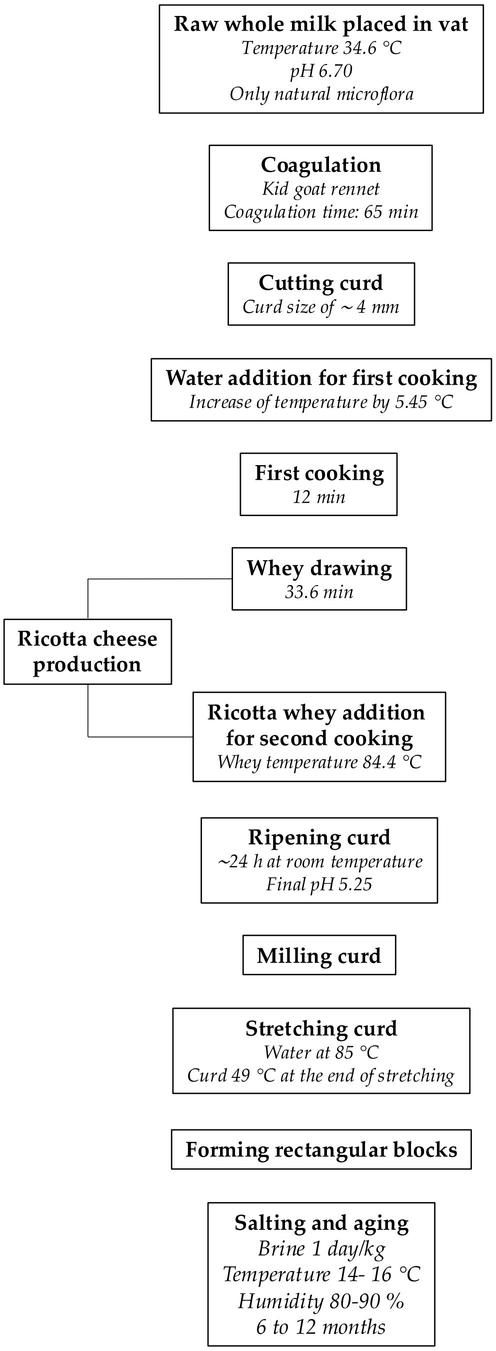


**Figure S1.** Flow chart of the Ragusano cheese-making process.
